# Temporomandibular Joint Bone Tissue Resorption in Patients with Early Rheumatoid Arthritis Can Be Predicted by Joint Crepitus and Plasma Glutamate Level

**DOI:** 10.1155/2010/627803

**Published:** 2010-06-29

**Authors:** Anna-Kari Hajati, Karin Näsström, Per Alstergren, Johan Bratt, Sigvard Kopp

**Affiliations:** ^1^Division of Clinical Oral Physiology, Department of Dental Medicine, Karolinska Institutet, P.O. Box 4064, 141 04 Huddinge, Sweden; ^2^Division of Oral Radiology, Department of Dental Medicine, Karolinska Institutet, P.O. Box 4064, 141 04 Huddinge, Sweden; ^3^Department of Rheumatology, Karolinska University Hospital, 141 86 Huddinge, Sweden

## Abstract

The aim was to investigate whether bone tissue resorption in early RA is related to crepitus of the temporomandibular joint (TMJ) and systemic levels of inflammatory mediators and markers and sex steroid hormones. Twentynine women and 18 men with recently diagnosed RA were examined for TMJ bone erosions with computerized tomography and TMJ crepitus was assessed. Blood samples were analyzed for glutamate, 5-HT, TNF, IL-1*β*, IL-6, VEGF, inflammatory markers, and estradiol, progesterone and testosterone. The TMJ erosion score was positively correlated to glutamate, and TMJ crepitus where crepitus, glutamate and ESR explained 40% of the variation in the bone erosion score. In the patients without crepitus, bone erosion score was positively correlated to glutamate, which was not the case in the patients with crepitus. In conclusion, the results of this study show that TMJ bone tissue resorption can be predicted by TMJ crepitus and glutamate in early RA.

## 1. Introduction

The main pathophysiology in rheumatoid arthritis (RA) is found in the synovial tissues of peripheral joints as an extensive infiltration of immunocompetent cells and high levels of inflammatory mediators. One common outcome is that inflammatory pannus tissue invades the cartilage and bone surface and causes cartilage and bone tissue destruction. This process results in joint crepitus, which is considered to be a clinical sign of joint surface destruction in chronic arthritides that is correlated to radiographic such as cortical erosions in the temporomandibular joint (TMJ) and hand joints [1, 2]. Crepitus in knee osteoarthritis has been found to be the strongest clinical indicator of radiographic changes in osteoarthritis (OA) [3]. However, another significant part of bone tissue resorption takes place within the subchondral bone marrow as a response to the systemic inflammation and mediators from the blood circulation [4]. Histologic studies of joint replacement specimens have shown that bone marrow resorptions with inflammatory cellular infiltrates [5] can be detected on MRI in 39%–75% of RA patients with disease duration of less than 1 year [6]. This was further shown in chronic RA patients subjected to joint replacement surgery in finger joints comparing immunohistochemistry with MRI [7]. 

We recently showed that TMJ bone tissue resorption is associated with plasma levels of glutamate, especially in patients with low-systemic inflammatory activity and low level of estradiol or testosterone [8]. The mechanisms behind bone tissue resorption observed in the TMJ in early RA may thus not be entirely related to systemic inflammation but also to other factors such as hormones. The question was therefore raised whether the glutamate-associated bone tissue resorption observed in the TMJ in early RA is associated with bone tissue destruction involving inflammatory mediators such as serotonin (5-HT) and proinflammatory cytokines or whether it is associated with noninflammatory mechanisms. 

Glutamate participates in normal bone turn over and bone tissue remodeling [9, 10] The glutamate level has been reported to be elevated in plasma [11] and synovial tissues in RA [12], probably as a result of release from activated thrombocytes [13, 14]. Continuous thrombocyte activation in the synovial tissues might be accomplished by proinflammatory cytokines such as tumor necrosis factor (TNF) since thrombocytes express cytokine receptors [15].

Serotonin may be involved in bone tissue resorption and the blood level of 5-HT has been related to erosions of TMJ bone tissue in patients with RA [16, 17]. 5-HT, which is mainly released from locally activated thrombocytes [18], is present in the bone marrow [19, 20] as well as the synovial membrane [21], synovial fluid [22], and blood plasma [23] of patients with RA. How glutamate and 5-HT are related is not known.

The sex steroid hormones estradiol, progesterone and testosterone take part in bone metabolism. Estradiol is known to suppress inflammation by inhibiting TNF [24, 25] and to increase the expression of osteoprotegerin [26] and thereby reduce bone resorption. The beneficial effects of estradiol on bone and inflammation may be neutralized and even antagonized by progesterone [27, 28]. Testosterone enhances bone production [25, 28]. 

The aim of this study was to investigate whether TMJ bone tissue resorption in early RA is related to gender or proinflammatory mediators or markers or the sex steroid hormones depending on whether there are clinical signs of crepitus or not in the TMJ. 

specific questions.

1. Is bone resorption of the TMJ related to crepitus as a clinical sign of articular cartilage and bone tissue damage?
2. Is bone resorption in patients with or without crepitus related to glutamate, 5-HT, cytokines (TNF, IL-1, IL-6, VEGF), CRP, ESR, RF, estradiol, progesterone or testosterone?
3. Are these relationships influenced by gender?

## 2. Material and Methods

### 2.1. Patients

Forty-seven patients, 29 females, and 18 males with RA were included in the study. Of these 28 (60%) were seropositive (Table 1). There was no statistically significant gender difference regarding the demographic or background variables investigated. The patients were consecutively invited to participate in the study after being diagnosed at the Department of Rheumatology, Karolinska University Hospital in Huddinge, Sweden. The inclusion criteria were diagnosis of RA according to the criteria of the American College of Rheumatology [29], age above 18, and verbal consent. Exclusion criteria were current malignancies, TMJ surgery or trauma within 2 years, recent intra-articular glucocorticoid injection in the TMJ (within 1 month), and conditions other than RA causing orofacial pain. 

This project was approved by the regional ethical committee at Karolinska Institutet (03-204) and by the local radiation committee at Karolinska University Hospital in Huddinge (19/03).

### 2.2. Medication

Eighty-one percent of the patients were on methotrexate treatment, which was initiated shortly before they entered the study at the time of diagnosis. Sixty-two percent used nonsteroidal anti-inflammatory drug medication, 44% received glucocorticoids, either by oral or intra-articular administration and 9% used salazopyrine or received gold salt injection. Two female patients used estradiol supplementation.

### 2.3. Clinical Protocol

The patients entered this study a median (25th–75th percentile) of 23 (12–36) days after diagnosis. They were examined clinically for joint crepitus, blood samples were collected and radiographs were taken during the same day. TMJ crepitus was assessed bilaterally as absent (0), palpable (1), or audible (2) and thus with an individual maximum score of 4. 

The disease activity score comprising 28 joints (DAS28) was assessed at the Department of Rheumatology, Karolinska University Hospital, Huddinge, Sweden, at the time of diagnosis.

### 2.4. Radiographic Examination

Radiographic examination of the TMJ region was performed with a cone-beam computer tomograph (CBCT; NewTom QR DVT mod 9000, Verona, Italy). The NewTom allowed a three-dimensional digital volume representation of the TMJ region, enabling sections of this region in any desired plane. The examinations were performed with a standardized head position with the TMJ located in the centre of the region of interest. The maximum size of the region of interest was 130 × 130 × 130 mm and scanning time was 70 seconds. Reconstructions were processed and analyzed by the NewTom software (NewTom 3G). The CBCT sections were evaluated for presence of erosions, that is, radiographic signs of bone tissue resorption. Erosion was defined as a local area with decreased mineral density of the articular cortical bone surface including or not including the adjacent subcortical bone or as subcortical bone tissue resorption under intact cortical bone. The erosions were assessed in 12 different regions of the condyle and temporal component of each TMJ. The number of TMJ bone erosions was counted (0–24) for each individual. The intraobserver consistency between repeated readings of bone erosion was tested on the images of 20 randomly selected joints from 10 of the patients. The scoring system was tested in a blinded fashion by the same radiologist at two occasions 2-3 years apart. On an individual level there was a total agreement in seven patients (70%) and a difference by one score unit in the other three patients.

### 2.5. Blood Sampling and Laboratory Procedures

In order to assess the inflammatory activity the inflammatory markers ESR (normal range: ♂ ≤ 70 years: ≤15 mm/h and ♂ > 70 years: ≤20 mm/h, ♀ ≤ 70 years: ≤20 mm/h and ♀ > 70 years: ≤28 mm/h), CRP (normal range: <3 mg/L), RF (normal range: <15 IU), thrombocyte particle count (TPC; normal range: ♂145–348 ∗10^9^/L, ♀  165–387 ∗10^9^/L), leukocyte particle count (LPC; normal range: ≤8.8 ∗10^9^/L), and anticyclic citrullinated peptide antibody titer (anti-CCP) (normal range: <25 U/mL) were analyzed in serum. Levels of estradiol, progesterone and testosterone were also determined in serum. All these analyses except for erythrocyte sedimentation rate were performed with standard procedures at the Department of Laboratory Medicine (Clinical Chemistry and Immunology), Karolinska University Hospital, Huddinge, Sweden. Determination of the erythrocyte sedimentation rate was performed according to the Westergren procedure. 

Venous blood sampled in EDTA tubes was immediately cooled and centrifuged (1500 g for 10 min at +4°C) for analysis of glutamate, TNF, IL-1*β*, and IL-6 in plasma. Other venous blood samples were stored without additives in room temperature for 1 hour to coagulate thereafter centrifuged (1500 g for 30 min at +4°C) for analyses of 5-HT serum levels. VEGF was analysed in lyzed whole blood. After preparation, the supernatants without cells were pipetted into polypropylene tubes except for serum samples that were stored in polystyrene tubes in −80°C until analysis. 

The concentrations of all investigated mediators were determined by commercially available assays in our laboratory. Glutamate in plasma was analysed by the Amplex Red Glutamic acid/glutamate oxidase assay (Amplex, Invitrogen, Eugene, USA). This glutamate assay had a detection limit of 10 *μ*mol/L according to the manufacturer. No information was given by the manufacturer about intra- and interassay variation. The median (5th–95th percentile) plasma level of glutamate in 22 healthy individuals (mean age 30 years, range 20–54 years) was found to be 6.2 (0.3–18) *μ*mol/L in our laboratory. 

TNF, IL-1*β*, and IL-6 in plasma were analysed with the high-sensitivity human cytokine Lincoplex kit for simultaneous multianalyte detection with Luminex technology and instrumentation (Lincoplex kit, Linco Research, St Charles, USA). The assay minimum detection concentration is 0.05 (TNF), 0.06 (IL-1*β*), and 0.10 (IL-6) pg/mL, respectively. The intra-assay coefficients of variation are 3.5% (TNF), 3.1% (IL-1*β*), and 3.5% (IL-6) and the inter-assay coefficient are 3.8% (TNF), 2.2% (IL-1*β*), and 4.5% (IL-6). The median (5th–95th percentile) levels of TNF (*n* = 30), IL-1*β* (*n* = 30) and IL-6 (*n* = 30) in a healthy control group have been reported to be 3.3 (1.2–7.8), 0.33 (0.0–2.1), and 187 (0.0−60) pg/mL, respectively [30]. The healthy group referenced comprised 48 subjects (23 men and 25 women; mean age 53 years, range 39–69 years).

VEGF was analysed by a commercially available enzyme-linked immunoassay kit (Quantikine Human Immunoassays, R&D Inc, Minneapolis, USA) in lysed whole blood. The median (range) VEGF level in normal lysed whole blood is 298 (92 to 554) pg/mL [31].

5-HT was analyzed by a commercially available competitive enzyme immunoassay kit (EIA-kit. No 0642, Immunotech International, Marseille, France). The kit has a detection limit and sensitivity of 0.5 nmol/L. The intra-assay coefficient of variation is less than 9.4% and the interassay coefficient of variation is less than 9.9%, according to the manufacturer. Normal serum 5-HT has a median (25th–75th percentile) of 602 (339−957) nmol/L, for males 626 (271−1819; *n* = 19, age = 35 (27−41) and for females 882 (437−1666) nmol/L (*n* = 35, age = 39 (30−51) according to our data. The levels are not correlated to age (*r*_*s*_ = −0,112, *n* = 52, *P* = .427).

Blood samples were also drawn to determine the serum levels of estradiol, progesterone and testosterone, according to standard procedures at the Department of Laboratory Medicine (Clinical Chemistry and Immunology), Karolinska University Hospital, Huddinge, Sweden. The normal reference range according to the laboratory for estradiol levels in fertile women was 100–1500 pmol/L, in postmenopausal women <50 pmol/L and in men 50–150 pmol/L. The normal reference intervall for progesterone levels in fertile women was at follicular phase <4.8 nmol/L and luteal phase >17 nmol/L and in postmenopausal women <3.0 nmol/L. In men the normal progesterone interval was <3.0 nmol/L. The range for testosterone levels in women was <2.7 nmol/L and in men 6–30 nmol/L.

### 2.6. Statistics

Median, min, max, and the 25th and 75th percentiles as well as mean and standard deviation were used for descriptive statistics. Correlations between the variables were tested in all patients as well as prespecified subgroups according to gender and TMJ crepitus by the Spearman rank correlation coefficient (*r*_*s*_). In order to select the strongest independent predictors to TMJ bone erosion score a stepwise linear multiple regression analysis was performed. Interaction analysis was performed by including an interaction term in addition to the main effects in the multiple linear regression model. The significance of differences according to gender and crepitus was tested with the Mann-Whitney *U*-test. A probability level of less than.05 was considered significant.

## 3. Results

The demographic and background variables are shown in Table 1. The levels of the inflammatory mediators as well as the bone erosion and crepitus scores are shown in Table 2. 

Bone erosion score was positively correlated to glutamate (*r*_*s*_ = 0.30, *P* = .042) and TMJ crepitus (*r*_*s*_ = 0.38, *P* = .008), while glutamate and TMJ crepitus were not significantly related (*r*_*s*_ = −0.08, *P* = .590).

**Figure 1:**
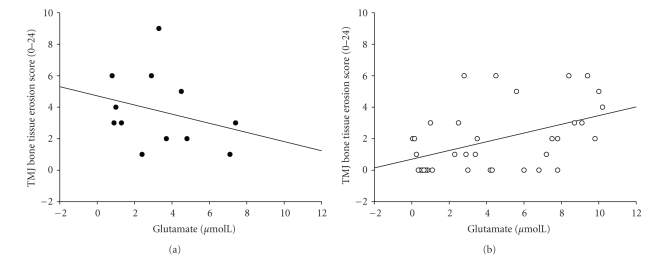
Scatter plots showing (a) the relationship between plasma level of glutamate and temporomandibular joint (TMJ) bone erosion score in the patients with crepitus (*r*_*s*_ = −0.35, *n* = 12, *P* = .253) and (b) in the patients with no crepitus (*r*_*s*_ = 0.45, *n* = 35, *P* = .007). ∙: crepitus, ∘: no crepitus.

Multiple stepwise regression showed that the independent variables TMJ crepitus (*P* < .001), glutamate (*P* = .005), and ESR (*P* = .010) were positively related to and explained 41% of the variation in the dependent variable TMJ bone erosion score (*P* < .001). Gender, 5-HT, TNF, IL-1, IL-6, VEGF, estradiol, progesterone, testosterone, RF, CRP, and ESR were also included into the stepwise regression but did not significantly add to the ability of the model to predict erosion score. In the patients without TMJ crepitus, bone erosion score was positively correlated to glutamate (*r*_*s*_ = 0.45, *n* = 35, *P* = .007; Figure 1). The corresponding correlation in the patients with crepitus was negative but did not reach statistical significance (*r*_*s*_ = −0.35, *n* = 12, *P* = .264; Figure 1). Interaction analysis with multiple linear regression showed that there was a different relation (*P* = .050) between bone erosion score and glutamate level in the patients without crepitus compared to those with crepitus.

**Figure 2:**
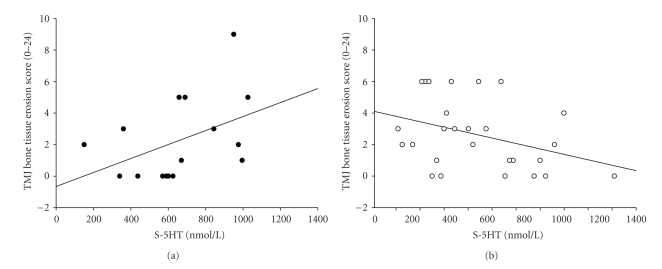
Scatter plots showing (a) the relationship between serum level of serotonin and temporomandibular joint (TMJ) bone erosion score in the male patients (*r*_*s*_ = 0.54, *n* = 16, *P* = .031) and (b) in the female patients (*r*_*s*_ = −0.34, *n* = 27, *P* = .080). ∙: males, ∘: females.

The bone erosion score was positively correlated to one of the investigated mediators of inflammation, 5-HT (*r*_*s*_ = 0.54, *n* = 16, *P* = .030; Figure 2), in males. The corresponding correlation in the females was negative but did not reach statistical significance (*r*_*s*_ = −0.34, *n* = 27, *P* = .080; Figure 2). Interaction analysis with multiple linear regression showed a significant (*P* = .014) interaction between gender and 5-HT. This implies a significant difference in relation between bone erosion and 5-HT in males compared to females.

## 4. Discussion

This study shows that glutamate is related to articular bone tissue resorption in the TMJ of patients with early RA without joint crepitus and that the presence bone resorption can be predicted by presence of joint crepitus and plasma level of glutamate. 

The crepitus in this study is mainly palpable (21%) and infrequently audible (2%), which indicates a lower degree of tissue destruction in agreement with the short disease duration. The frequency of crepitus was lower in this study than in a previous study where 75% of both male and female RA patients were reported to have crepitus [32]. However, the majority of these patients had disease duration of more than 5 years. Another study reported a weak but statistically significant relationship between crepitus and radiologic changes of the TMJ in RA patients, where 80% of joints with crepitus showed evidence of articular bone erosion while only 47% of joints with radiologic erosion had crepitus [2]. This latter finding is in agreement with ours and indicates that articular bone erosions develop with different mechanisms. The regression model also included ESR as a predictor of bone resorption when the other variables were considered. ESR was not significantly correlated to the bone erosion score in the non-parametric univariate analysis and this relationship should therefore be interpreted with great caution. However, such a relation can be expected for erosions of systemic inflammatory nature and strong association between serial measurements of ESR and radiographic joint destruction has been found [33].

The relationship between bone tissue resorption and serum level of 5-HT seems to be positive and specific for men since there was a significant gender difference regarding this relationship. Plasma and serum levels of 5-HT have been reported to be associated with progression of TMJ bone resorption during a 25–46 month interval in patients with established RA [17], indicating a systemic influence by 5-HT on local bone tissue resorption. 5-HT may modulate osteoblast bone formation via the 5-HT_2B_ receptor [11, 34, 35] and the osteoclast differentiation through its transporter 5-HTT [19]. The exact nature of the 5-HT contribution to bone tissue resorption and the influence of gender is, however, still unclear. In this study, the observed bone tissue resorption cannot be considered as normal remodeling due to the combination with active RA. However, 5-HT may play an important role for articular bone tissue remodeling in both healthy individuals and in patients with RA since osteocytes and osteoblasts express receptors for 5-HT and 5-HTT [36]. A longitudinal study of mainly female patients with chronic RA remarkably showed that low-serum levels of 5-HT were associated with progression of erosions, while high levels were associated with regression [17]. Whether glutamate or 5-HT is associated with continuing bone tissue resorption in the early stage of RA is not yet known.

In this study, no dietary precautions like fasting were taken before blood sampling and quantification of 5-HT and glutamate. The plasma level of glutamate found in this study is lower than that reported from a previous study of RA patients [8] as well as in a sample of healthy individuals investigated in our clinic. A significantly higher nonfasting level of glutamate was found in the RA patients in comparison with healthy individuals [8]. This previous study deviates from ours regarding age and disease duration of the patients. However, since no relation was observed between glutamate level and age nor disease duration in our patient sample [11], the discrepancies between the studies may be of methodological nature.

The patients included had a recently diagnosed RA and most of them were examined for this study within less than one month after diagnosis and about 6 months after their report of debut of symptoms. Most patients commenced anti-inflammatory medication shortly after diagnosis and before the start of this investigation. An influence of the medication on the levels of inflammatory markers and mediators is likely but the influence of the medication on the radiographic signs of bone tissue resorption can be expected to be minor or even insignificant.

In conclusion, the results of this study show that presence of bone tissue resorption of the TMJ can be predicted by presence of a combination of joint crepitus and elevated plasma level of glutamate in early RA. The relation between glutamate and TMJ bone tissue resorption is different in patients with or without crepitus indicating that glutamate has a particular role in joints without surface destruction. The results also show that bone tissue resorption at this early stage is related to the inflammatory mediator 5-HT in men but not in women.

## Figures and Tables

**Table 1:** Demographic and background data for 47 patients with early rheumatoid arthritis.

				Percentiles							
			Median	25th	75th	Min	Max	Mean	SD	*n*	% abn
Gender		M/F								18/29	
Age		years	62	53	67	25	79	58	14	47	
Duration of general joint symptoms		months	7	4	10	2.3	38	8	6	47	
Duration of RA diagnosis		days	23	12	36	3	115	27	20	47	
Anti-cyclic citrullinated peptide		IU/mL	30	0	453	0	2000	349	557	37	51
Rheumatoid factor		IU/mL	23	0	173	0	910	130	221	47	51
C-reactive protein		Mg/L	0	0	18	0	96	13	22	47	43
Erythrocyte sedimentation rate		mm/h	22	17	32	6	85	28	18	47	49
Leukocyte particle count		10^9^/L	7.5	5.6	8.7	4.3	15	7.8	2.7	39	54
Thrombocyte particle count		10^9^/L	315	273	356	148	657	331	99	47	23
Disease activity score (DAS28)		0–10	6	5	6.5	4	7.8	6	1	44	100

Estradiol	total	pmol/L	62	34	82	15	1299	111	213	42	
	males	pmol/L	65	62	85	48	140	77	25	17	
	females	pmol/L	41	26	66	15	1299	133	275	25	

Testosterone	total	nmol/L	1.2	0	10	0.0	22	5.0	6.1	42	
	males	nmol/L	12	7.9	14	4.3	22	11	4.5	17	
	females	nmol/L	0	0	0.9	0.0	3.5	0.6	0.8	25	

Progesterone	total	nmol/L	0.4	0	1	0.0	2.1	0.6	0.6	42	
	males	nmol/L	0.9	0.0	1.2	0.0	1.8	0.8	0.6	17	
	females	nmol/L	0.0	0.0	0.7	0.0	2.1	0.4	0.6	25	

% abn: percentage of observations with abnormal values when applicable, *n*: number of observations, M: males, F: tamales, IU: international units. The following values ware considered abnormal: Anti-cyclic citrullinated peptide ≥25 U/mL, rheumatoid tactor ≥20 IU, C- reaclive protein ≥3 mg/L, erythrocyte sedimentation rate >24 mm/h, leukocyte particle count >8.8 × 10^9^/L, and thrombocyte particle count ♂ > 348 × 10^9^/L and ♀ > 387 × 10^9^/L. The disease activity score for 28 joints (DAS28) was assessed at the time for diagnosis.

**Table 2:** Erosion score as well as level of TMJ erosion score and inflammatory mediators in blood samples from 47 patients with early rheumatoid arthritis.

			Percentile							
		Median	25th	75th	Min	Max	Mean	SD	*n*	%abn
Erosion score	0–24	2.0	00	3.8	0	9	2.4	2.3	47	72
TMJ crepitus	0–4	0	0	0.8	0	4	0.3	0.7	47	26
Blood levels										
Plasma										
Glutamate	*μ*mol/L	3.4	1.0	7.2	0.1	10.2	4.1	3.2	47	
Tumor necrosis factor	Pg/mL	9	7.5	14	4.0	107	14	16	47	68
Interleukin-1*β*	pg/mL	3.3	1.1	7.7	0.5	46	5.2	7.1	47	64
Interleukin-6	pg/mL	27	14	36	2.8	200	32	32	47	36
Serum										
Serotonin	nmol/L	610	358	958	16.3	1818	667	395	43	
Whole blood										
Vascular endothelial growth factor	Pg/mL	143	99	186	46	1027	179	170	36	28

*n*: number of observations, % abn: % of patients with values outside the** 5**th–95th percentile range according to the reference values (see Section 2).
